# Global, regional, and national burden of multiple myeloma from 1990 to 2021 and projections for 2040: a systematic analysis for the global burden of disease 2021 study

**DOI:** 10.3389/fepid.2025.1568688

**Published:** 2025-04-29

**Authors:** Yuying Wei, Wenjuan Gao, Shuai Wang, Qizhao Li, Shuqian Xu

**Affiliations:** Department of Hematology, Qilu Hospital of Shandong University, Jinan, Shandong, China

**Keywords:** multiple myeloma, age-standardized rates, incidence, mortality, disability-adjusted life years

## Abstract

**Background:**

Multiple myeloma (MM) is a malignant hematologic disorder characterized by the abnormal clonal proliferation of bone marrow plasma cells and excessive production of immunoglobulins, often leading to severe organ damage. Due to its high incidence, recurrence, and death rates, MM poses a significant burden on individuals and global healthcare systems. This study leverages the latest data from the Global Burden of Disease Study 2021 (GBD 2021) to analyze the epidemiological trends of MM and propose effective preventive strategies.

**Methods:**

Using data from GBD 2021, we analyzed the age-standardized incidence rate (ASIR), death rate (ASDR), and disability-adjusted life years (DALYs) of MM, evaluating temporal trends through estimated annual percentage change (EAPC). Pearson correlation analysis was employed to explore the relationship between age-standardized rates (ASRs) and the Sociodemographic Index (SDI). Additionally, frontier analysis was conducted. Finally, Bayesian age-period-cohort models were utilized to predict the trends of MM ASRs through 2040.

**Results:**

In 2021, the global number of new MM cases was 148,755 (95% UI: 131,780.4–162,049.2), with 116,359.6 deaths (95% UI: 103,078.6–128,470.6) and 2,595,595 DALYs (95% UI: 2,270,483.6–2,889,968.2). Age-standardized rates increased with age. Between 1990 and 2021, the global burden of MM exhibited a consistent upward trend across all populations, with males and older adults bearing the highest burden. The analysis demonstrated a positive correlation between ASRs and the SDI. Frontier analysis indicated regions with medium-to-high SDI have the greatest potential for reducing ASRs. Among all risk factors, high body mass index (BMI) was identified as the most significant contributor to MM. Projections suggest that by 2040, the global burden of MM may experience a decline.

**Conclusion:**

Driven by population aging and advancements in diagnostic capabilities, the global burden of multiple myeloma continues to rise. Targeted prevention and treatment strategies, particularly for elderly and high-risk populations, are essential to alleviate the disease burden and improve patient outcomes.

## Introduction

Multiple myeloma (MM) is a malignant hematologic disease characterized by the clonal proliferation of plasma cells in the bone marrow, leading to the production of large quantities of monoclonal immunoglobulin (M-protein). It is commonly associated with severe organ damage, including renal failure, osteolytic bone lesions, anemia, and hypercalcemia (1). MM predominantly affects elderly individuals, with the median age at diagnosis being 69 years, making it the second most common hematologic malignancy. MM accounts for 10% of all hematologic malignancies and approximately 20% of related mortality. The incidence of MM has been steadily increasing globally, resulting in a heavy health burden (2–4). The pathogenesis of MM is linked to multiple risk factors, including obesity, chronic inflammation, radiation exposure, and genetic susceptibility (5). Although recent advances in novel targeted therapies and autologous stem cell transplantation (ASCT) have prolonged patient survival (6, 7), the progression and relapse of the disease, driven by subclonal evolution and drug resistance, along with a shortened duration of remission (1), continue to present significant challenges. Ultimately, patients succumb to the disease itself or treatment-related complications, and the global burden of MM remains substantial. Previous studies on the epidemiological characteristics of MM have primarily relied on earlier versions of the Global Burden of Disease (GBD) data or focused on localized analyses within specific regions or countries (8–10). However, significant differences exist in the burden and trend of MM across different regions, countries, and socio-economic groups. Accurate, real-time epidemiological data on MM is crucial for reducing its incidence and alleviating the burden on healthcare systems. To address these limitations, we utilized the latest GBD 2021 database to conduct a more in-depth data mining and complex analysis. This study provides the most recent evaluation of the trends in MM incidence, mortality, and Disability-Adjusted Life Years (DALYs) from 1990 to 2021 at global, regional, and national levels. Additionally, we performed gender, age, and Socio-Demographic Index (SDI) stratified analyses, as well as an attribution analysis of risk factors. We also conducted a cutting-edge, forward-looking analysis to project the future burden of MM, offering valuable insights into understanding the disease. Our findings aim to provide significant guidance for the biology, prevention, and treatment of MM, and establish a solid scientific foundation for the rational allocation of public health resources and the development of effective prevention strategies.

## Materials and methods

### Data source and collection

The data for this study were obtained from the GBD database, which compiles information from sources including vital registration systems, verbal autopsies, censuses, disease registries, and contributions from GBD collaborators, providing comprehensive estimates of the global disease burden. The GBD 2021 study employed the most recent epidemiological data and standardized methods to comprehensively assess health losses associated with 371 diseases, injuries, and conditions, and 88 risk factors, across 21 regions and 204 countries and territories from 1990 to 2021 (11). We extracted data on MM from the Global Health Data Exchange (GHDx) query tool, which offers estimates of incidence, mortality, and DALYs globally, regionally, and nationally, stratified by age and sex, and analyzed health outcome data from 1990 to 2021.

DALY is a key metric for evaluating the burden of disease, representing the total number of years lost due to ill health, disability, or premature death. The Socio-Demographic Index (SDI) combines fertility rate, education level, and per capita income to quantify a country or region's socio-economic development level (12), categorizing nations and regions into five groups: low, low-middle, middle, middle-high, and high. SDI is used to assess the relationship between socio-economic development and the burden of MM (13).

### Statistical analyses

This study analyzed the age-standardized rates (ASR) of incidence, mortality, and DALYs for MM at global, regional, and national levels. To assess the temporal dynamics of the ASR, a logarithmic linear regression model, *y* = *α* + *βx* + *ε* [where *y* = ln (ASR), *x* = calendar year, and *ε* is the error term], was employed. The annualized percentage change (EAPC) was calculated using the formula EAPC = 100 × [exp (*β*)—1], and statistical significance was evaluated using 95% confidence intervals (CI). If the lower bound of the EAPC 95% CI is above zero, the ASR is defined as increasing; if the upper bound is below zero, the ASR is defined as decreasing; otherwise, the ASR is considered stable. Spearman's correlation analysis was used to assess the relationship between ASR and SDI. Frontier analysis was employed to assess the potential for improving health metrics across countries at their current SDI levels. This was achieved by estimating the theoretical minimum ASR attainable under each country's SDI and comparing it with the observed ASR, thereby quantifying the room for improvement in health outcomes (14). In addition, we conducted an attribution analysis of MM based on risk factor data available in the database. To project future trends in the MM burden, we adopted the Bayesian Age-Period-Cohort (BAPC) model to fit data from 1990 to 2021 and forecast ASRs through 2040. Compared with conventional models such as ARIMA and Joinpoint regression, the BAPC model offers superior performance in analyzing data with complex structures and age–period interactions, providing more robust and reliable projections. Model fitting and forecasting were implemented using the BAPC (version 0.0.36) and INLA (version 23.09.09) packages in R, and 95% uncertainty intervals (UI) were reported to assess the reliability of the predicted results.

Data processing, statistical analysis, and visualization in this study were conducted using R software (version 4.3.2), with specific packages including BAPC, ggplot2, and dplyr. Descriptive statistics were generated for all key variables, with results presented as means and 95% UI or 95% CI. For trend analysis, *p*-values less than 0.05 were considered statistically significant. All analyses in this study were based on publicly available GBD database data, and since the original research was ethically approved, no additional ethical approval or informed consent was required.

## Results

### Global burden of MM

**Figure 1:**
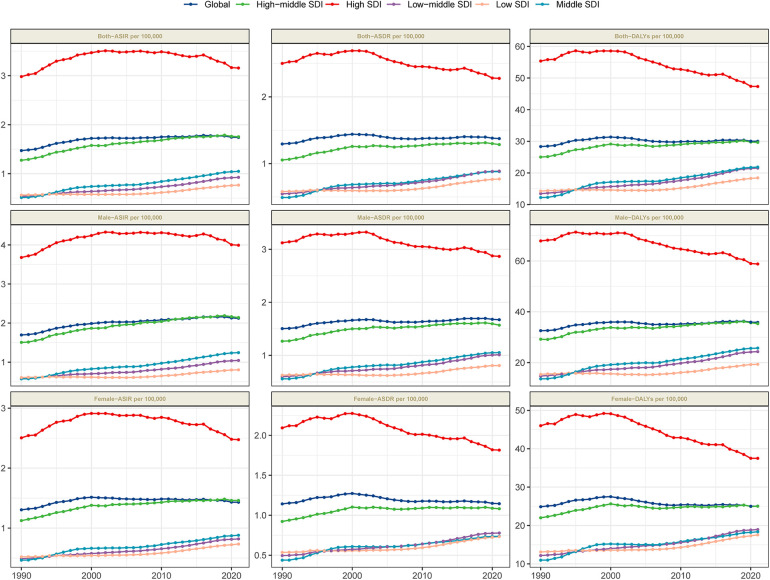
Trends in MM incidence, mortality, and disability-adjusted life years from 1990 to 2021.

By 2021, the global burden of MM remained substantial. The number of new cases increased from 55,710.1 (95% UI: 52,022.5–59,687.8) in 1990 to 148,754.6 (95% UI: 131,780.4–162,049.2) in 2021. The age-standardized incidence rate (ASIR) rose slightly from 1.5 per 100,000 population (95% UI: 1.4–1.6) in 1990 to 1.7 per 100,000 (95% UI: 1.5–1.9) in 2021, with an EAPC of 0.48 (95% CI: 0.37–0.6), indicating a gradual increase in incidence over the study period (Table 1, Figure 1). In 2021, MM was responsible for an estimated 116,359.6 deaths (95% UI: 103,078.6–128,470.6), with an age-standardized death rate (ASDR) of 1.4 per 100,000 population (95% UI: 1.2–1.5). The EAPC for ASDR was 0.15 (95% CI: −0.02 to 0.33), reflecting a slow rise in mortality, potentially attributable to advancements in treatment (Table 1 and Figure 1). MM also caused a significant number of DALYs, amounting to 2,595,595 (95% UI: 2,270,483.6–2,889,968.2) in 2021. The age-standardized DALY rate was 30 per 100,000 population (95% UI: 26.2–33.4), with an EAPC of 0.06 (95% CI: −0.04 to 0.15), indicating a gradual increase in the burden of premature mortality and reduced quality of life (Table 1 and Figure 1).

**Table 1:** Global and regional trends in MM burden: incidence, mortality, and disability-adjusted life years (1990–2021).

Location	1990		2021		EAPC (95% CI)
	Number	ASR	Number	ASR	
Incidence					
Global	55,710.1 (52,022.5–59,687.8)	1.5 (1.4–1.6)	148,754.6 (131,780.4–162,049.2)	1.7 (1.5–1.9)	0.48 (0.37–0.6)
High SDI	33,358.1 (31,614.9–34,445)	3 (2.8–3.1)	68,287.5 (61,342.1–72,525.4)	3.2 (2.9–3.3)	0.15 (−0.02 to 0.33)
High-middle SDI	12,561.7 (11,834–13,538.5)	1.3 (1.2–1.4)	34,787.5 (30,245.1–38,625.2)	1.8 (1.5–1.9)	1.01 (0.89–1.13)
Middle SDI	5,249.2 (4,561.2–6,753.4)	0.5 (0.4–0.7)	28,497.7 (22,906.1–33,491.5)	1 (0.8–1.2)	2.15 (1.98–2.33)
Low-middle SDI	3,221.8 (2,332.5–4,254.8)	0.5 (0.4–0.7)	13,200.8 (11,292.7–18,482.7)	0.9 (0.8–1.3)	1.72 (1.64–1.8)
Low SDI	1,243.3 (695.7–1,749.9)	0.6 (0.3–0.8)	3,801 (2,523.3–5,130.7)	0.8 (0.5–1)	0.95 (0.78–1.13)
Andean Latin America	231.7 (170.4–295.1)	1.1 (0.8–1.5)	1,061 (824.7–1,379.2)	1.8 (1.4–2.3)	1.59 (1.4–1.78)
Australasia	986.9 (920.9–1,058.1)	4.2 (3.9–4.5)	2,991.1 (2,604.8–3,386)	5.5 (4.8–6.2)	1.03 (0.9–1.17)
Caribbean	615.1 (568.1–677)	2.4 (2.2–2.6)	1,711.6 (1,464.2–1,960.8)	3.2 (2.7–3.6)	0.98 (0.86–1.1)
Central Asia	137.5 (118.6–154.1)	0.3 (0.2–0.3)	440.2 (392.2–492.6)	0.5 (0.4–0.6)	2.42 (2.03–2.81)
Central Europe	2,181.1 (2,046.8–2,297.3)	1.4 (1.3–1.5)	4,952.5 (4,505.7–5,371.3)	2.2 (2–2.4)	1.36 (1.16–1.56)
Central Latin America	924.1 (894.6–949.7)	1.1 (1.1–1.1)	4,143.9 (3,699.1–4,654.5)	1.6 (1.5–1.8)	1.15 (1.05–1.26)
Central Sub-Saharan Africa	81 (53.9–109.4)	0.4 (0.2–0.5)	239.6 (131.7–345.2)	0.4 (0.2–0.6)	0.68 (0.4–0.96)
East Asia	1,918.1 (1,370–3,616.1)	0.2 (0.2–0.4)	18,188.6 (11,881.7–23,583.1)	0.8 (0.5–1.1)	3.88 (3.25–4.51)
Eastern Europe	2,943.9 (2,788.6–3,117.7)	1 (1–1.1)	6,170 (5,703.1–6,689.9)	1.8 (1.6–1.9)	1.9 (1.64–2.15)
Eastern Sub-Saharan Africa	644.7 (351.1–923.8)	0.9 (0.5–1.3)	2,059.3 (1,252.6–2,823.4)	1.2 (0.8–1.7)	1.06 (0.94–1.17)
High-income Asia Pacific	3,969.4 (3,680.5–4,198.1)	2 (1.8–2.1)	9,740.9 (8,163.3–10,907.1)	1.9 (1.7–2.2)	−0.04 (−0.22 to 0.14)
High-income North America	11,849.7 (11,190.6–12,239.4)	3.3 (3.2–3.4)	20,898.2 (19,023.8–22,011)	3.1 (2.8–3.3)	−0.39 (−0.54 to −0.24)
North Africa and Middle East	1,370.4 (953.7–1,860.8)	0.8 (0.6–1.1)	5,840.4 (4,386.5–8,004.5)	1.3 (1–1.8)	1.6 (1.51–1.69)
Oceania	9.1 (5.6–13.3)	0.3 (0.2–0.5)	26.1 (14.9–37)	0.4 (0.2–0.5)	0.28 (0.24–0.33)
South Asia	3,537.5 (2,253.9–4,500.7)	0.6 (0.4–0.8)	15,905.4 (12,551.4–21,561.9)	1.1 (0.9–1.5)	1.62 (1.46–1.78)
Southeast Asia	755.6 (601.1–1,226.1)	0.3 (0.2–0.5)	3,507.8 (2,721.4–5,680.9)	0.5 (0.4–0.9)	1.8 (1.75–1.85)
Southern Latin America	1,027.9 (954.4–1,113.8)	2.2 (2.1–2.4)	2,033.2 (1,875.6–2,190.1)	2.3 (2.2–2.5)	0.25 (0.08–0.41)
Southern Sub-Saharan Africa	403.3 (276–521.3)	1.5 (1–1.9)	1,351.4 (890.9–1,639.5)	2.3 (1.5–2.8)	1.49 (1.36–1.63)
Tropical Latin America	1,200.3 (1,149.4–1,245.4)	1.3 (1.2–1.3)	5,412.5 (5,051–5,693.1)	2.1 (2–2.2)	1.49 (1.32–1.66)
Western Europe	20,706.3 (19,518.5–21,531.4)	3.5 (3.4–3.7)	41,185.2 (36,907.1–44,033.1)	4.3 (3.9–4.6)	0.64 (0.43–0.86)
Western Sub-Saharan Africa	216.5 (112.5–301)	0.3 (0.1–0.4)	895.8 (366–1,315.9)	0.5 (0.2–0.7)	2.15 (2.01–2.29)
Deaths					
Global	47,569 (44,137.5–51,416.5)	1.3 (1.2–1.4)	116,359.6 (103,078.6–128,470.6)	1.4 (1.2–1.5)	0.09 (−0.01 to 0.18)
High SDI	28,142.7 (26,550.8–28,971.1)	2.5 (2.4–2.6)	51,434.8 (45,704.7–54,830.5)	2.3 (2.1–2.4)	−0.43 (−0.54 to −0.32)
High-middle SDI	10,133.5 (9,535–10,990.2)	1.1 (1–1.1)	25,451.4 (22,131.8–28,174.5)	1.3 (1.1–1.4)	0.59 (0.48–0.71)
Middle SDI	4,837.8 (4,187.4–6,267.9)	0.5 (0.4–0.6)	23,404.8 (18,799.8–27,500.9)	0.9 (0.7–1)	1.72 (1.54–1.91)
Low-middle SDI	3,156 (2,278.9–4,161.6)	0.5 (0.4–0.7)	12,282.8 (10,526.8–17,215.5)	0.9 (0.8–1.2)	1.53 (1.46–1.61)
Low SDI	1,235.2 (688.7–1,741.5)	0.6 (0.3–0.8)	3,648.5 (2,427.4–4,903.8)	0.8 (0.5–1)	0.87 (0.7–1.03)
Andean Latin America	223.5 (164.8–281.8)	1.1 (0.8–1.4)	883.1 (692.8–1,147.2)	1.5 (1.2–2)	1.08 (0.91–1.25)
Australasia	670.4 (628.7–708.8)	2.8 (2.7–3)	1,656.9 (1,438–1,854.8)	2.9 (2.5–3.2)	0.12 (0.01–0.24)
Caribbean	465.3 (433.1–517.1)	1.8 (1.7–2)	1,109.5 (957.2–1,256.4)	2.1 (1.8–2.3)	0.45 (0.36–0.55)
Central Asia	126.8 (110–141.9)	0.3 (0.2–0.3)	386.9 (344.4–434.2)	0.5 (0.4–0.5)	2.28 (1.91–2.64)
Central Europe	2,028.9 (1,908.1–2,141)	1.3 (1.3–1.4)	4,421.7 (4,024.9–4,787.7)	1.9 (1.8–2.1)	1.08 (0.91–1.25)
Central Latin America	843.6 (817.7–865.9)	1 (1–1.1)	3,331.2 (2,986.4–3,732.1)	1.3 (1.2–1.5)	0.73 (0.64–0.82)
Central Sub-Saharan Africa	79.9 (53.4–107.8)	0.4 (0.3–0.5)	227.3 (123.9–329)	0.4 (0.2–0.6)	0.59 (0.32–0.85)
East Asia	1,763.5 (1,244.3–3,362.1)	0.2 (0.1–0.4)	13,624.9 (9,018.1–17,740)	0.6 (0.4–0.8)	2.99 (2.31–3.68)
Eastern Europe	2,459.2 (2,333.6–2,585.3)	0.9 (0.8–0.9)	4,652.4 (4,296–5,043)	1.3 (1.2–1.4)	1.51 (1.29–1.72)
Eastern Sub-Saharan Africa	642.7 (352.1–919.1)	0.9 (0.5–1.3)	1,974.2 (1,210.4–2,699)	1.2 (0.8–1.7)	0.96 (0.85–1.07)
High-income Asia Pacific	3,029.8 (2,836.3–3,195)	1.5 (1.4–1.6)	6,975.3 (5,830.4–7,775.2)	1.3 (1.1–1.4)	−0.77 (−0.9 to −0.64)
High-income North America	11,712.8 (10,989.6–12,106.6)	3.3 (3.1–3.4)	19,376.8 (17,536.4–20,470.3)	2.8 (2.6–3)	−0.67 (−0.79 to −0.56)
North Africa and Middle East	1,289.9 (897.8–1,753)	0.8 (0.6–1.1)	4,708.3 (3,544.4–6,487.8)	1.1 (0.8–1.5)	1.1 (1.02–1.17)
Oceania	8.4 (5.2–12.4)	0.3 (0.2–0.5)	23.8 (13.5–34.4)	0.3 (0.2–0.5)	0.21 (0.16–0.25)
South Asia	3,476.2 (2,220.5–4,424.9)	0.6 (0.4–0.8)	14,791 (11,658.6–20,033.1)	1 (0.8–1.4)	1.41 (1.26–1.55)
Southeast Asia	702.9 (557.9–1,149.1)	0.3 (0.2–0.5)	2,985.8 (2,308.4–4,846.6)	0.5 (0.4–0.8)	1.5 (1.44–1.55)
Southern Latin America	957.5 (890.9–1,033.5)	2.1 (1.9–2.2)	1,675.3 (1,544.4–1,791.3)	1.9 (1.7–2)	−0.22 (−0.38 to −0.06)
Southern Sub-Saharan Africa	385.9 (262.8–502.7)	1.5 (1–1.9)	1,235.4 (817.1–1,492.8)	2.2 (1.4–2.6)	1.35 (1.19–1.51)
Tropical Latin America	1,096.9 (1,047.2–1,136.1)	1.2 (1.2–1.3)	4,585.7 (4,254.1–4,822.7)	1.8 (1.7–1.9)	1.24 (1.09–1.39)
Western Europe	15,385.5 (14,492.5–15,919.1)	2.6 (2.4–2.7)	26,874.7 (23,758.8–28,878.2)	2.6 (2.3–2.8)	0 (−0.13 to 0.12)
Western Sub-Saharan Africa	219.4 (113.5–305.8)	0.3 (0.1–0.4)	859.3 (363.2–1,242.6)	0.5 (0.2–0.7)	2.01 (1.89–2.13)
Disability-adjusted life years					
Global	11,22,517.3 (10,41,399.5–12,27,728.7)	28.3 (26.3–30.8)	25,95,595 (22,70,483.6–28,89,968.2)	30 (26.2–33.4)	0.06 (−0.04 to 0.15)
High SDI	609,781.3 (585,948.4–625,027.7)	55.4 (53.3–56.7)	976,932.5 (896,756.9–10,33,833.1)	47.3 (44–49.8)	−0.64 (−0.76 to −0.52)
High-middle SDI	2,54,005.9 (2,39,764.5–2,77,110.1)	25 (23.6–27.3)	5,83,311.9 (5,04,141.2–6,52,355)	29.7 (25.5–33.2)	0.47 (0.37–0.56)
Middle SDI	1,35,857.9 (1,17,165.7–1,75,791.4)	12.2 (10.6–15.8)	6,09,118.6 (4,87,413.4–7,14,664.8)	21.8 (17.5–25.6)	1.68 (1.49–1.87)
Low-middle SDI	87,031.8 (62,726.6–1,15,403)	13.5 (9.7–17.7)	3,23,359 (2,74,506.6–4,49,769.1)	21.5 (18.3–30)	1.49 (1.42–1.56)
Low SDI	34,293 (18,917.4–48,485.5)	14.3 (8–20.1)	99,828 (66,163.6–1,36,137.1)	18.4 (12.3–24.9)	0.75 (0.59–0.91)
Andean Latin America	5,896.1 (4,316.8–7,408.1)	27.8 (20.4–34.8)	22,215.6 (17,273.3–29,040.8)	37 (28.8–48.2)	1.04 (0.86–1.22)
Australasia	14,960.8 (14,101.4–15,747.6)	63.6 (60–67)	32,239.5 (28,598.2–35,867.7)	60.6 (54.1–67.4)	−0.08 (−0.17 to 0.01)
Caribbean	11,307.3 (10,445.9–12,749.8)	43 (39.8–48.4)	26,898.8 (23,074–30,706.5)	49.9 (42.8–56.9)	0.52 (0.42–0.62)
Central Asia	4,070.6 (3,526.8–4,563.3)	7.8 (6.8–8.8)	11,936.9 (10,635.4–13,424.2)	13.1 (11.6–14.6)	2.06 (1.73–2.4)
Central Europe	50,815.1 (47,917.3–53,407.4)	33.2 (31.3–34.9)	95,122.8 (86,993.4–1,03,200.3)	43.9 (40.1–47.7)	0.83 (0.66–1)
Central Latin America	23,525.5 (22,810–24,147.5)	26.4 (25.6–27.1)	88,782.2 (79,138.4–99,663.7)	34.4 (30.7–38.6)	0.73 (0.64–0.83)
Central Sub-Saharan Africa	2,296.3 (1,506.4–3,140.6)	9.4 (6.3–12.7)	6,648 (3,617.5–9,691.1)	10.9 (5.9–15.8)	0.58 (0.32–0.84)
East Asia	51,846.4 (37,024.8–98,327.4)	5.4 (3.8–10.2)	3,54,332.9 (2,28,713.8–4,64,089.7)	16.3 (10.4–21.4)	3.03 (2.38–3.67)
Eastern Europe	69,119.7 (65,361.7–72,840.4)	24.2 (22.9–25.6)	1,18,762.6 (1,09,042.8–1,28,799.4)	34.5 (31.7–37.4)	1.19 (1.01–1.37)
Eastern Sub-Saharan Africa	17,754.3 (9,542.9–25,618)	22.4 (12.3–32)	55,541.4 (33,194.2–77,262)	30.1 (18.3–41.2)	0.94 (0.83–1.05)
High-income Asia Pacific	69,220.4 (65,580.4–72,653.7)	33.9 (32–35.6)	1,18,265.9 (1,01,403.6–1,30,303.1)	25.6 (22.5–28)	−1.05 (−1.19 to −0.9)
High-income North America	2,52,724.9 (2,42,624.1–2,59,512.5)	73.5 (70.8–75.4)	3,74,036.8 (3,48,987.1–3,90,777.2)	57.3 (53.8–59.8)	−1.04 (−1.16 to −0.92)
North Africa and Middle East	35,663.8 (24,579.6–48,761)	19.8 (13.7–27)	1,24,880.9 (93,507.5–1,71,852.8)	26 (19.5–35.9)	0.96 (0.89–1.04)
Oceania	247.9 (149.7–369.3)	7.8 (4.8–11.5)	697.4 (384.4–1,013.4)	8.4 (4.8–12.2)	0.29 (0.24–0.34)
South Asia	97,101 (61,059.7–1,23,438.5)	15.8 (10.1–20.1)	3,83,531.8 (3,02,576.4–5,11,789.8)	25 (19.7–33.4)	1.32 (1.17–1.46)
Southeast Asia	19,905.6 (15,810.5–32,175.9)	7.2 (5.8–11.8)	80,580.3 (62,894.1–1,28,420.2)	11.5 (9–18.6)	1.42 (1.36–1.48)
Southern Latin America	23,126.6 (21,634.8–24,929.3)	49.4 (46.1–53.2)	37,800.1 (35,378.2–40,287.5)	44 (41.2–46.9)	−0.31 (−0.47 to −0.15)
Southern Sub-Saharan Africa	10,830.1 (7,509.7–13,641.4)	37.3 (25.6–47.9)	34,716.8 (23,139.6–42,583.4)	55.5 (36.8–67.5)	1.38 (1.22–1.53)
Tropical Latin America	30,472.1 (29,402.7–31,534.5)	31.2 (30–32.4)	1,13,479.2 (1,07,328.9–1,18,279.7)	43.4 (41–45.3)	0.98 (0.82–1.14)
Western Europe	3,26,015.4 (3,11,080.9–3,36,759.7)	57.3 (54.9–59)	4,92,170.3 (4,47,363.6–5,23,560.6)	53.6 (49.6–56.6)	−0.25 (−0.39 to −0.11)
Western Sub-Saharan Africa	5,617.2 (2,902.4–7,816.8)	6.2 (3.2–8.7)	22,955 (9,456.9–33,911.5)	11.1 (4.7–16.1)	2.04 (1.91–2.18)

### The regional burden of MM

In 2021, the burden of MM varied significantly across regions, with incidence, mortality, and DALY levels closely correlated with the SDI. Among the five SDI groups, the high-SDI region bore the highest burden, with an ASIR of 3.2 per 100,000 (95% UI: 2.9–3.3), an ASDR of 2.3 per 100,000 (95% UI: 2.1–2.4), and DALYs of 47.3 per 100,000 (95% UI: 44–49.8), more than twice that of the low-SDI regions. All five SDI groups showed an increasing trend in ASIR (with positive EAPC values), while high-SDI regions exhibited a decreasing trend in ASDR and DALYs (with negative EAPC values) (Table 1 and Figure 1), indicating a reduction in mortality and disease burden over the study period.

Among the 21 GBD regions, Oceania had the lowest MM burden, with an ASIR of 0.4 per 100,000 (95% UI: 0.2–0.5), death rate of 0.3 per 100,000 (95% UI: 0.2–0.5), and DALYs ranging from 23.0 to 43.0 per 100,000 (Table 1). In contrast, Australasia had the highest burden, with an ASIR of 5.5 per 100,000 (95% UI: 4.8–6.2), an ASDR of 2.9 per 100,000 (95% UI: 2.5–3.2), and DALYs of 60.6 per 100,000 (95% UI: 54.1–67.4) (Table 1). Additionally, regions such as Western Europe and High-income North America also showed a significant burden of MM. The temporal trends of MM burden across different regions exhibited varied patterns. Except for the High-income Asia Pacific and High-income North America regions, the remaining 19 regions showed a consistent increase in incidence. Death rates decreased in High-income Asia Pacific, Southern Latin America, and High-income North America, while there was no significant change in Western Europe; the other 17 regions experienced an increase in mortality. Overall, the MM burden decreased at higher levels in Western Europe, High-income North America, High-income Asia Pacific, and Australasia, whereas it increased in the remaining regions, particularly East Asia, where the rise was most pronounced (Table 1).

### National burden of MM

**Figure 2:**
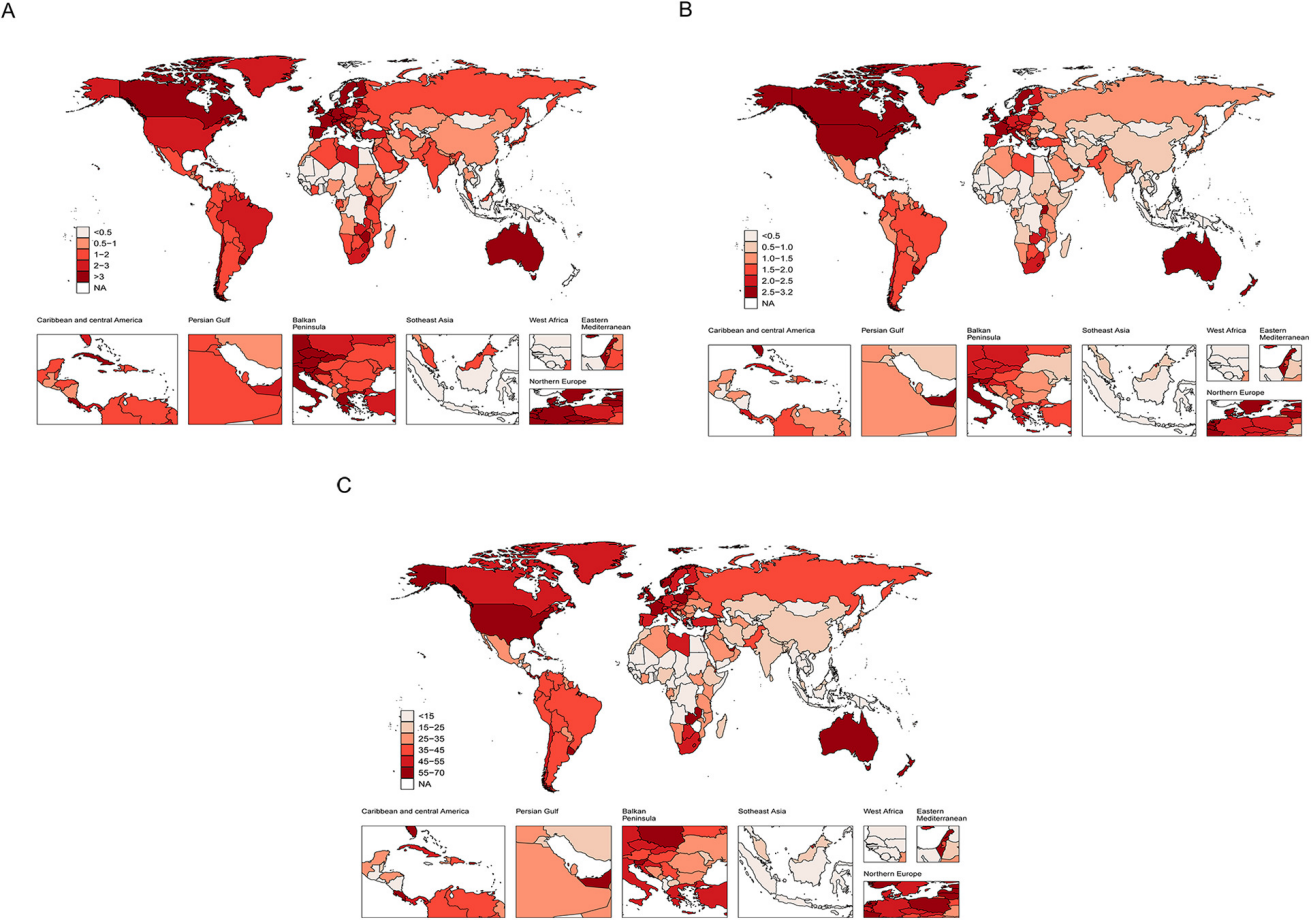
The global burden of MM in 204 countries and territories. **(A)** Age-standardized incidence rate. **(B)** Age-standardized death rate. **(C)** Age-standardized DALY rate.

The burden of MM varies significantly across countries and regions. Figure 2 and Supplementary Table S1 summarize the age-standardized metrics for MM in 2021, as well as the EAPC analysis from 1990 to 2021. In terms of ASIR in 2021, the countries with the highest incidence rates were Monaco (6.9 per 100,000; 95% UI: 3.5–10.9), Bahamas (6.5 per 100,000; 95% UI: 5.2–8.2), and New Zealand (6 per 100,000; 95% UI: 5.2–6.7). Georgia showed the largest increase in ASIR (EAPC: 6.2; 95% CI: 5.45–6.96) (Figure 2A, Supplementary Figure S1). Regarding ASDR in 2021, the top three countries were Bahamas (4.7 per 100,000; 95% UI: 3.9–5.7), Monaco (4.4 per 100,000; 95% UI: 2.3–6.8), and Grenada (3.6 per 100,000; 95% UI: 3.1–4). Georgia also showed the greatest increase in ASDR (EAPC: 6.18; 95% CI: 5.43–6.95) (Figure 2B, Supplementary Figure S1). The countries with the highest DALY burden in 2021 were the Bahamas (117.5 years per 100,000; 95% UI: 94.3–145.1), Monaco (93.3 years per 100,000; 95% UI: 47.4–150.1), and Zimbabwe (88.7 years per 100,000; 95% UI: 48.8–126.7). Turkmenistan had the largest increase in age-standardized DALYs from 1990 to 2021 (EAPC: 6.04; 95% CI: 5.45–6.64) (Figure 2C, Supplementary Figure S1). In 2021, both Bahamas and Monaco exhibited consistently high values across all three metrics, highlighting that both countries face extremely high MM burden.

### Disease burden of MM by sex and age

**Figure 3:**
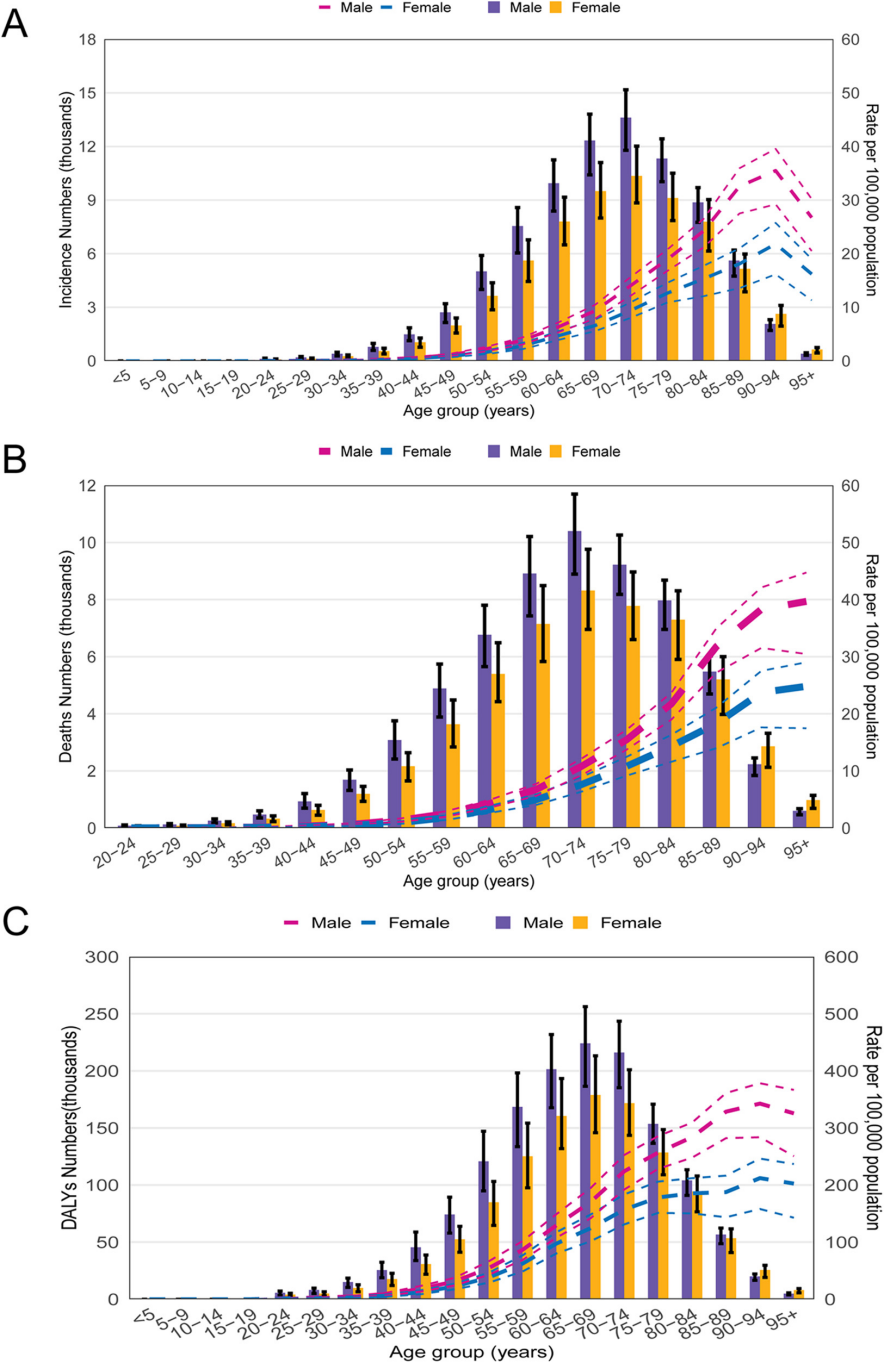
The global burden of MM by age group and gender in 2021. **(A)** The number of incident cases and incidence rate. **(B)** The number of deaths and death rate; **(C)** The number and rate of DALYs caused by MM.

Age and sex are critical parameters in the epidemiological study of MM. A global age- and sex-specific analysis in 2021 revealed that the incidence of MM increases markedly with age, exhibiting a similar upward trend in both males and females (Figure 3A). Notably, the incidence rises sharply among individuals aged 70 years and older, highlighting advanced age as a significant risk factor for MM. Given the ongoing global population aging trend, the MM's burden in this age group is expected to continue rising. Therefore, targeted interventions for the elderly population may be crucial for mitigating the growing global disease burden. Furthermore, the analysis showed that MM incidence rates were consistently higher in males than in females across all age groups, with the sex disparity widening with increasing age. This pattern suggests that genetic factors may play a key role in the pathogenesis of MM. Similar trends were observed in mortality and DALY rates, which also increased with age in both sexes. However, the disease burden was significantly higher in males than in females across all age groups (Figures 3B,C).

### Relationship between MM burden and SDI

**Figure 4:**
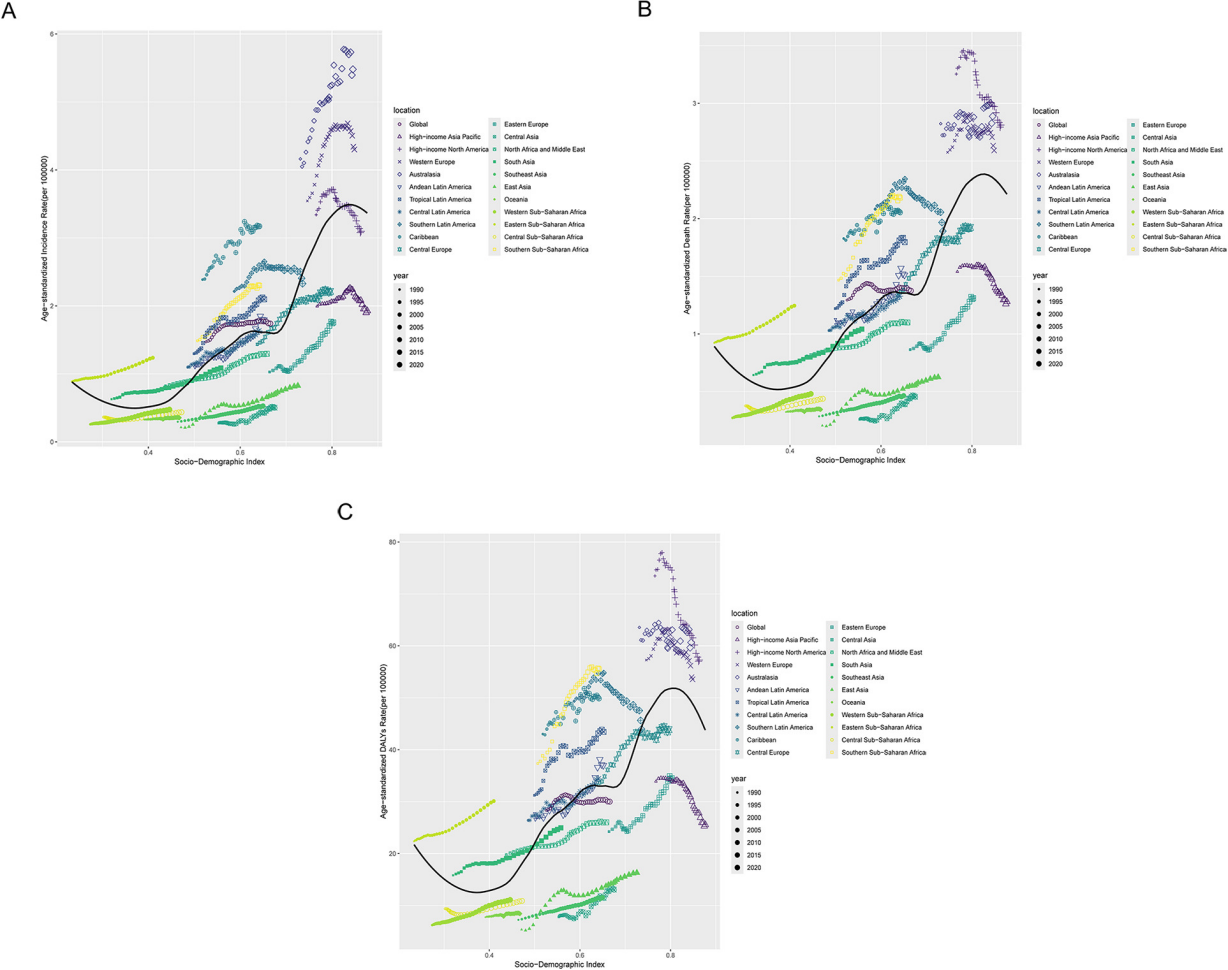
Correlation between socio-demographic Index (SDI) and MM burden across 21 GBD regions. **(A)** The incidence rate per 100,000 population; **(B)** the death rate per 100,000 population; **(C)** the DALY rate per 100,000 population.

Across the globe and within the 21 GBD regions, a non-linear relationship was observed between the SDI and the ASIR, ASDR, and DALYs of MM. When SDI was below 0.4, the MM burden tended to decrease with increasing SDI. In regions with SDI values between 0.4 and 0.8, the MM burden increased as SDI rose. However, in regions with SDI above 0.8, further increases in SDI were associated with a decline in MM burden. Notably, substantial decreases in MM burden were observed in high-income Asia Pacific, high-income North America, Western Europe, and Australasia (Figures 4A–C).

### Frontier analysis of MM burden

**Figure 5:**
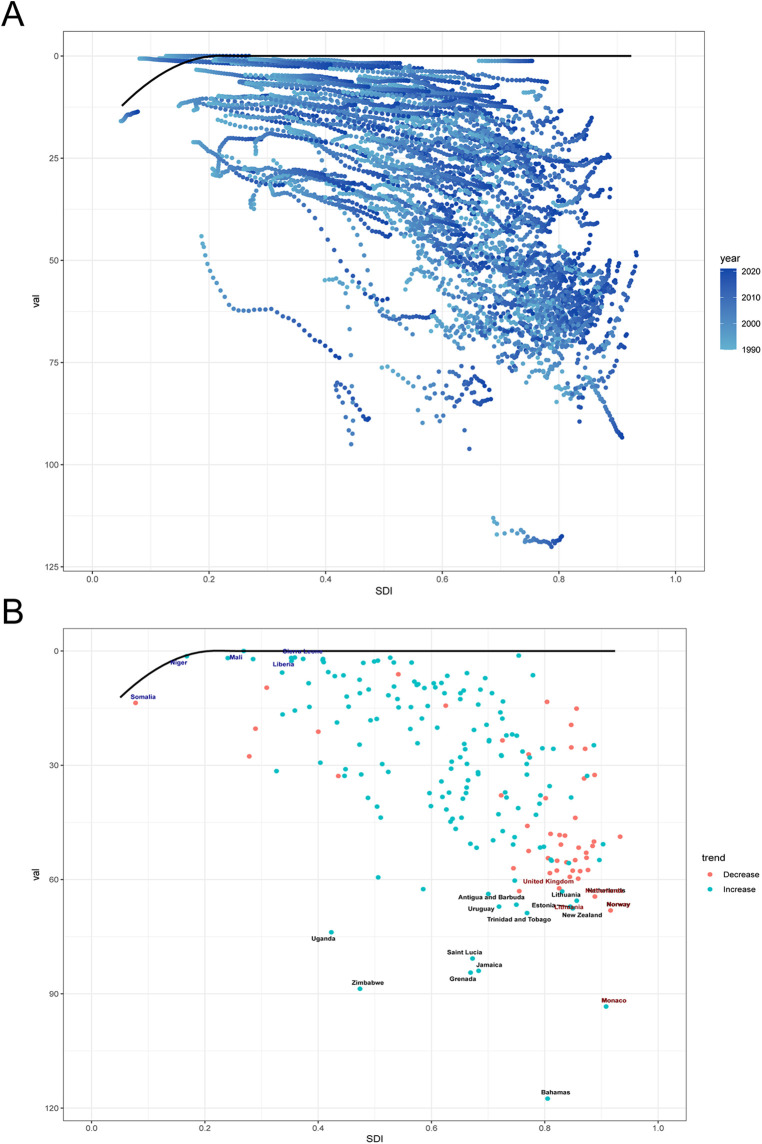
Frontier analysis based on SDI and age-standardized DALY rate of MM from 1990 to 2021. **(A)** The blue dots represent the age-standardized DALY rate, with darker shades indicating later years. **(B)** The dots represent changes in age-standardized DALY rate. Black lines represent the lower limits of ASR achievable at different SDI levels, with points representing different countries and regions. The 15 countries and regions with the largest effective differences globally are labeled in black font, the 5 countries and regions with the smallest effective differences among low SDI countries are labeled in blue font, and the 5 countries and regions with the largest effective differences among high SDI countries are labeled in red font.

Frontier analysis revealed that regions with higher SDI have greater potential for reducing the burden of MM compared to low-SDI regions (Figure 5A). Over time, high-SDI regions have achieved more substantial progress in alleviating MM burden, suggesting that they possess stronger healthcare resources and a greater capacity to implement effective interventions. Notably, the 15 countries with the highest MM burden are predominantly located in middle-high and high-SDI regions (Figure 5B). Considerable disparities in MM burden exist among these countries, indicating that despite progress in the healthcare sector, significant gaps still remain.

### Attributable risk factors for the MM burden

**Figure 6:**
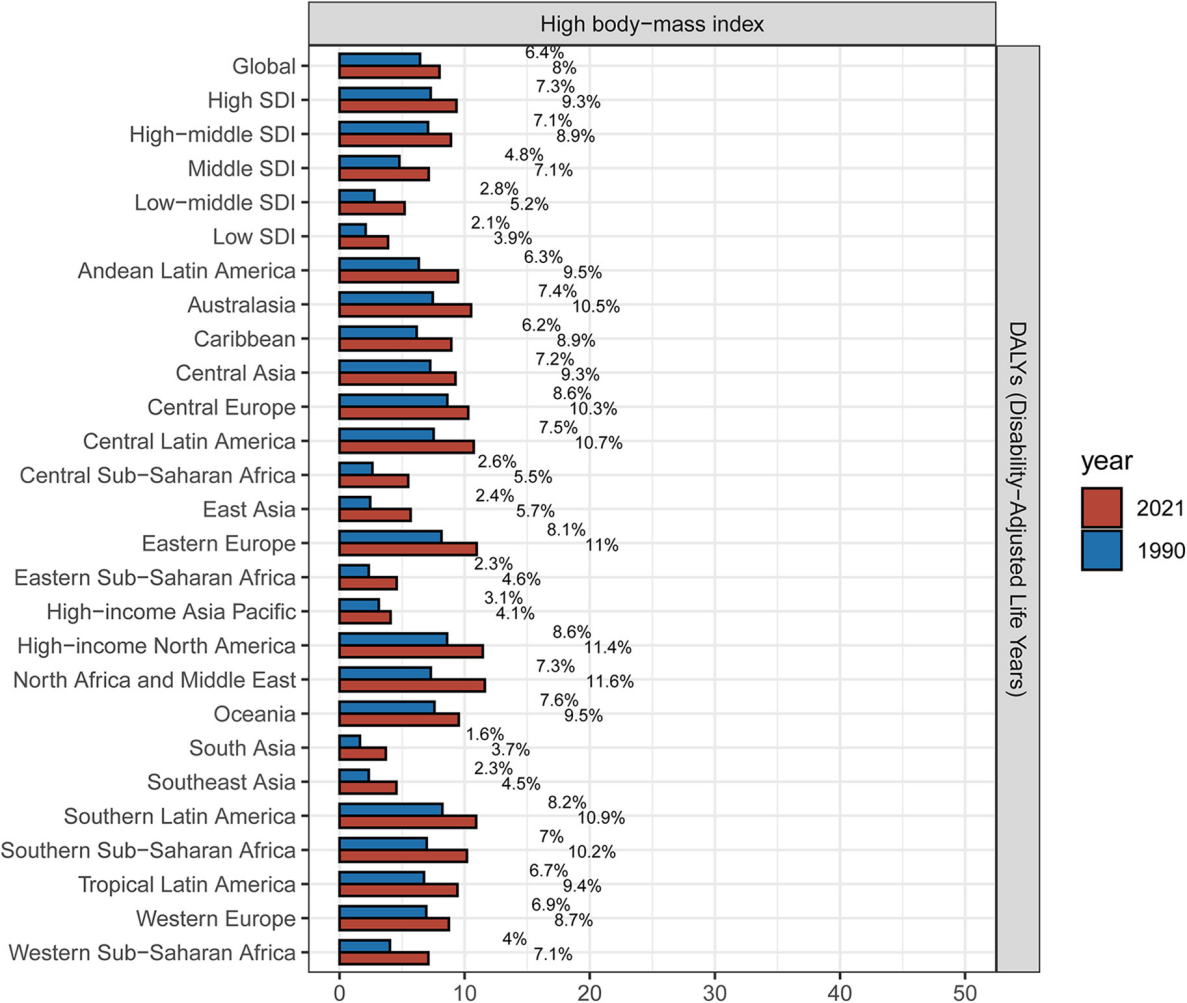
Proportion of MM DALYs attributed to HBMI in the global, 5 SDI zones, 21 GBD regions in 1990 and 2021.

Analysis of data from GBD 2021 identified high body mass index as the primary risk factor for the burden of MM. Compared to 1990, the proportion of MM burden attributable to HBMI increased globally and across all regions in 2021, with a global contribution rising by 1.6% (Figure 6). HBMI accounted for a larger share in high-SDI regions, North Africa and the Middle East, high-income North America, and Eastern Europe—areas identified in this study as having significant MM burden—highlighting the exacerbating impact of obesity on MM. Although the proportion of HBMI in low-SDI regions, South Asia, East Asia, and Eastern Sub-Saharan Africa remained relatively small, its upward trend indicates that the influence of obesity on MM is expanding. This underscores the urgent need to strengthen obesity prevention and management and implement targeted public health strategies to mitigate the impact of obesity on MM.

### Projections of the global burden of MM

**Figure 7:**
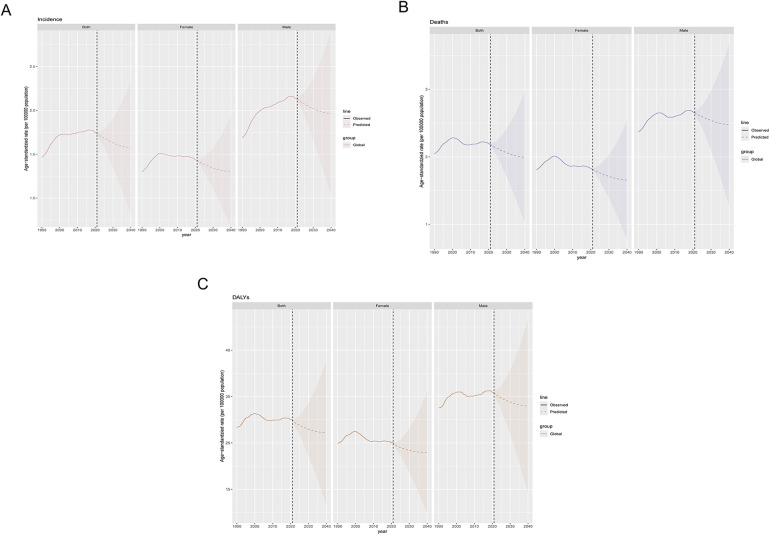
Predicted trends for MM incidence, mortality, and disability-adjusted life years from 1990 to 2040. **(A)** Incidence; **(B)** Mortality; **(C)** DALYs.

Projection analysis suggests that by 2040, the three age-standardized metrics used to measure the global burden of MM will follow similar decreasing trends in both sexes. The ASIR of MM is projected to decrease to 1.6 per 100,000 people (95% CI: 0.8–2.3) by 2040 (Figure 7A, Supplementary Figure S2). The ASDR is expected to decline to 2.0 per 100,000 people (95% CI: 1.0–3.0) (Figure 7B, Supplementary Figure S2). Additionally, the age-standardized DALY for MM is anticipated to decrease to approximately 27.1 years per 100,000 people (95% CI: 11.6–42.7) (Figure 7C, Supplementary Figure S2).

## Discussion

Our study utilized the GBD 2021 database to conduct a comprehensive and up-to-date analysis of the disease burden, risk factors, and future trends of MM at global, regional, and national levels, providing important evidence for future public health decisions and healthcare resource allocation. The study found that in 2021, there were 148,754.6 new cases of MM globally, with 116,359.6 deaths and a total of 2,595,595 years of DALYs lost. The ASR of incidence, mortality, and DALYs for MM showed an upward trend throughout the study period, indicating that the global burden of MM continues to rise each year. This trend is consistent with previous GBD studies (8, 15), and may be attributed to factors such as population aging, improved diagnostic capabilities, and advancements in treatment, all of which have led to increased survival. At the regional level, high-SDI regions such as Australasia, Western Europe, and High-income North America face a more severe disease burden. This may be attributed to factors such as more accurate diagnoses, advanced treatment methods, and the accelerating aging of the population in these regions (16). In contrast, low-SDI regions generally exhibit lower incidence and death rates, which may reflect limited healthcare infrastructure and underreporting of cases, resulting in a lower reported burden. This finding highlights the persistent socio-economic disparities in the global burden of MM. Notably, the EAPC values for MM indicators in Middle-SDI regions are the highest, indicating that the disease burden is rapidly increasing in these areas. This may be due to factors such as improved healthcare facilities during economic development transitions, population aging, and changes in environmental and lifestyle factors. Therefore, it is crucial to develop more personalized public health strategies for these countries and regions, focusing on enhancing early diagnosis and treatment of MM. At the national level, the countries with the heaviest burden of MM are Monaco and the Bahamas, both of which rank highly in terms of age-standardized indicators. Further investigation into the reasons for their high incidence is crucial for understanding the risk factors associated with MM. In addition, high-income countries such as Norway, New Zealand, and Australia also bear a substantial disease burden, although the trends have decreased compared to 1990. This is consistent with the findings from the regional analysis, suggesting that well-established healthcare systems and advanced treatment technologies in high-income countries have played a key role in reducing MM mortality and disease burden.

Our study further revealed significant differences in the burden of MM based on age and gender. The data show that with increasing age, the incidence, mortality, and DALYs of MM significantly rise, particularly in populations aged 60 and older. Additionally, the burden of MM is higher in males compared to females, with this gender disparity becoming more pronounced in older age groups. These findings are consistent with the known epidemiological patterns of MM (17). The higher risk of MM in males may be attributed to multiple factors, including genetic susceptibility, environmental risks, and occupational exposures (18).

The nonlinear relationship between the burden of MM and the SDI reveals the complex association between socioeconomic development and disease. When SDI is <0.8, the MM burden increases with rising SDI, indicating that in low-SDI regions, economic development is often accompanied by underdeveloped public health systems, thereby exacerbating the burden. However, when SDI >0.8, the disease burden shows a downward trend, particularly in regions such as High-income Asia Pacific, High-income North America, Western Europe, and Australasia. This trend is closely linked to the advanced healthcare systems and disease prevention measures in high-SDI regions. This finding is consistent with the broader association between SDI and the burden of MM at the regional level. It indicates that, although substantial progress has been made in reducing the MM burden in high-SDI regions, middle- and low-SDI regions still face numerous serious challenges. These challenges stem from inequitable distribution of healthcare resources, including shortages of medical professionals, insufficient public awareness, delayed diagnosis, limited access to novel therapies, and the high cost of treatment (19–21). The frontier analysis provided new insights into the potential future trajectory of the MM burden. By analyzing the data, we identified the 15 most promising countries located in upper-middle SDI regions. This finding underscores the need for policymakers to implement targeted public health strategies to mitigate the future impact of MM.

We conducted an attribution analysis of the risk factors associated with MM in the GBD database and identified high body mass index as a primary risk factor for MM. Increasing evidence supports obesity as a significant modifiable risk factor for MM (22, 23), with MM recognized as obesity-related cancer (OAC) (24, 25). A higher body mass index is associated with an increased risk of MM, more rapid disease progression, and poorer prognosis (26–28). Previous studies have suggested that obesity may contribute to the initiation and progression of MM through various mechanisms, including the upregulation of pro-inflammatory cytokines, dysregulation of adipokine secretion, and impaired immune surveillance (23, 29, 30). Obesity is an escalating global health issue, and as obesity rates increase worldwide, the burden of MM is also rising. Therefore, implementing public health initiatives focused on weight management, promoting lifestyle changes such as low-salt, low-fat diets, and enhancing physical activity is of paramount importance for preventing MM before onset, improving prognosis, and enhancing patients' quality of life to reduce the disease burden.

This study presents the first prediction analysis of the global MM burden from 2022 to 2040, providing valuable insights for policy development, long-term healthcare investments, planning, and priority setting. The predictive analysis suggests that the global MM burden may show a slight downward trend over the next 20 years, particularly in high-income countries. In recent years, the combination of chemotherapy with hematopoietic stem cell transplantation, immunomodulatory agents, proteasome inhibitors, monoclonal antibodies, bispecific antibodies, and CAR-T therapies has significantly extended survival and reduced death rates (31, 32), offering hope for improved outcomes. However, this trend is not universally observed, and the MM burden in low- and middle-income regions may continue to rise, particularly in areas with limited healthcare resources and low public health standards. Therefore, it is critical to develop region-specific public health policies and improve early diagnosis and treatment resource allocation, especially in aging societies and resource-limited regions, to address the future MM burden effectively.

However, our study also has some limitations. First, the accuracy of the estimates may be influenced by the quality and availability of data from different countries and regions. In low-SDI areas, the burden of MM may be underestimated due to limited healthcare resources, lower diagnostic rates, and incomplete reporting mechanisms. Second, due to data constraints, we were unable to fully assess the impact of other potential risk factors, such as environmental exposures and metabolic abnormalities, on the MM burden. Further analysis of these factors is needed in future research.

## Conclusion

This study utilized the GBD 2021 database to systematically evaluate the global, regional, and national burden of MM and to project future trends. The results showed that from 1990 to 2021, the global ASIR, ASDR, and DALYs for MM increased steadily. The burden was particularly pronounced in high-SDI regions, males, and the elderly, reflecting the combined effects of population aging, unequal healthcare resource distribution, and genetic factors. Although the burden in high-SDI regions has slightly decreased, it remains at a relatively high baseline level. In contrast, the burden in low-SDI regions may be underestimated due to insufficient resources and underreporting. Over the next two decades, the MM burden is projected to decline slightly in high-income countries but may continue to rise in low-income countries, particularly in regions with aging populations and limited healthcare resources. These findings provide essential evidence for informing public health policies, optimizing resource allocation, and refining clinical strategies for MM. However, future efforts should focus on improving data accuracy and exploring the underlying causes of regional disparities.

## Data Availability

The original contributions presented in the study are included in the article/Supplementary Material, further inquiries can be directed to the corresponding author.

## References

[1] van de Donk N Pawlyn C Yong KL. Multiple myeloma. Lancet. (2021) 397:410–27. 10.1016/S0140-6736(21)00135-5 33516340

[2] Cowan AJ Green DJ Kwok M Lee S Coffey DG Holmberg LA Diagnosis and management of multiple myeloma: a review. JAMA. (2022) 327:464–77. 10.1001/jama.2022.0003 35103762

[3] Malard F Neri P Bahlis NJ Terpos E Moukalled N Hungria VTM Multiple myeloma. Nat Rev Dis Primers. (2024) 10(1):45. 10.1038/s41572-024-00529-7 38937492

[4] Rajkumar SV. Multiple myeloma: 2024 update on diagnosis, risk-stratification, and management. Am J Hematol. (2024) 99:1802–24. 10.1002/ajh.27422 38943315 PMC11404783

[5] Kazandjian D. Multiple myeloma epidemiology and survival: a unique malignancy. Semin Oncol. (2016) 43:676–81. 10.1053/j.seminoncol.2016.11.004 28061985 PMC5283695

[6] Nishimura KK Barlogie B van Rhee F Zangari M Walker BA Rosenthal A Long-term outcomes after autologous stem cell transplantation for multiple myeloma. Blood Adv. (2020) 4:422–31. 10.1182/bloodadvances.2019000524 31990333 PMC6988393

[7] Rajkumar SV. Multiple myeloma: 2022 update on diagnosis, risk stratification, and management. Am J Hematol. (2022) 97:1086–107. 10.1002/ajh.26590 35560063 PMC9387011

[8] Cowan AJ Allen C Barac A Basaleem H Bensenor I Curado MP Global burden of multiple myeloma: a systematic analysis for the global burden of disease study 2016. JAMA Oncol. (2018) 4:1221–7. 10.1001/jamaoncol.2018.2128 29800065 PMC6143021

[9] Curado MP Oliveira MM Silva DRM Souza DLB. Epidemiology of multiple myeloma in 17 Latin American countries: an update. Cancer Med. (2018) 7:2101–8. 10.1002/cam4.1347 29573332 PMC5943416

[10] Dou X Duan G Zhong Y Liu Y Peng N Wen L The burden of multiple myeloma in China: trends from 1990 to 2021 and forecasts for 2050. Cancer Lett. (2025) 611:217440. 10.1016/j.canlet.2025.217440 39755360

[11] Ferrari AJ Santomauro DF Aali A Abate YH Abbafati C Abbastabar H Global incidence, prevalence, years lived with disability (YLDs), disability-adjusted life-years (DALYs), and healthy life expectancy (HALE) for 371 diseases and injuries in 204 countries and territories and 811 subnational locations, 1990–2021: a systematic analysis for the global burden of disease study 2021. Lancet. (2024) 403:2133–61. 10.1016/S0140-6736(24)00757-8 38642570 PMC11122111

[12] Wang H Abbas KM Abbasifard M Abbasi-Kangevari M Abbastabar H Abd-Allah F Global age-sex-specific fertility, mortality, healthy life expectancy (HALE), and population estimates in 204 countries and territories, 1950–2019: a comprehensive demographic analysis for the global burden of disease study 2019. Lancet. (2020) 396:1160–203. 10.1016/S0140-6736(20)30977-6 33069325 PMC7566045

[13] Li H-Z Liang X-Z Sun Y-Q Jia H-F Li J-C Li G. Global, regional, and national burdens of osteoarthritis from 1990 to 2021: findings from the 2021 global burden of disease study. Front Med. (2024) 11:1476853. 10.3389/fmed.2024.1476853 PMC11602326 39610688

[14] Sultana S Hossain MM Haque MN. Estimating the potato farming efficiency: a comparative study between stochastic frontier analysis and data envelopment analysis. PLoS One. (2023) 18:e0284391. 10.1371/journal.pone.0284391 37053255 PMC10101415

[15] Zhou L Yu Q Wei G Wang L Huang Y Hu K Measuring the global, regional, and national burden of multiple myeloma from 1990 to 2019. BMC Cancer. (2021) 21:606. 10.1186/s12885-021-08280-y 34034700 PMC8152089

[16] Velez R Turesson I Landgren O Kristinsson SY Cuzick J. Incidence of multiple myeloma in Great Britain, Sweden, and Malmo, Sweden: the impact of differences in case ascertainment on observed incidence trends. BMJ Open. (2016) 6:e009584. 10.1136/bmjopen-2015-009584 26801465 PMC4735168

[17] Padala SA Barsouk A Barsouk A Rawla P Vakiti A Kolhe R Epidemiology, staging, and management of multiple myeloma. Med Sci. (2021) 9(1):3. 10.3390/medsci9010003 PMC7838784 33498356

[18] Huang J Chan SC Lok V Zhang L Lucero-Prisno DE 3rd Xu W The epidemiological landscape of multiple myeloma: a global cancer registry estimate of disease burden, risk factors, and temporal trends. Lancet Haematol. (2022) 9:e670–7. 10.1016/S2352-3026(22)00165-X 35843248

[19] Chng WJ Nagarajan C Huang SY Malhotra P Hwang YY Blunk V A systematic review on the epidemiology and treatment options of multiple Myeloma in Asia. Heliyon. (2024) 10:e39698. 10.1016/j.heliyon.2024.e39698 39553611 PMC11566861

[20] Mattar M Bazarbachi A Abduljalil O Francis B Alam A Blunk V. Epidemiology, treatment trends, and outcomes of multiple Myeloma in the Middle East and Africa: a systematic review. Clin Hematol Int. (2024) 6:67–83. 10.46989/001c.92555 38817690 PMC11086989

[21] Thokerunga E Ntege C Ahmed AO. Are African primary physicians suspicious enough? Challenges of multiple myeloma diagnosis in Africa. Egypt J Intern Med. (2021) 33:54. 10.1186/s43162-021-00088-3

[22] Tentolouris A Ntanasis-Stathopoulos I Terpos E. Obesity and multiple myeloma: emerging mechanisms and perspectives. Semin Cancer Biol. (2023) 92:45–60. 10.1016/j.semcancer.2023.04.003 37030643

[23] Tie W Ma T Yi Z Liu J Li Y Bai J Obesity as a risk factor for multiple myeloma: insight on the role of adipokines. Pathol Oncol Res. (2023) 29:1611338. 10.3389/pore.2023.1611338 37637774 PMC10447903

[24] Lauby-Secretan B Scoccianti C Loomis D Grosse Y Bianchini F Straif K Body fatness and cancer–viewpoint of the IARC working group. N Engl J Med. (2016) 375:794–8. 10.1056/NEJMsr1606602 27557308 PMC6754861

[25] Wang L Xu R Kaelber DC Berger NA. Glucagon-like peptide 1 receptor agonists and 13 obesity-associated cancers in patients with type 2 diabetes. JAMA Netw Open. (2024) 7:e2421305. 10.1001/jamanetworkopen.2024.21305 38967919 PMC11227080

[26] Arnold KD Ong KL Ravi G Cutshall H Purnell K Wessel MC Anthropometric traits and risk of multiple myeloma: differences by race, sex and diagnostic clinical features. Br J Cancer. (2024) 131:312–24. 10.1038/s41416-024-02723-6 38849476 PMC11263363

[27] Birmann BM Andreotti G De Roos AJ Camp NJ Chiu BCH Spinelli JJ Young adult and usual adult body mass index and multiple myeloma risk: a pooled analysis in the international multiple myeloma consortium (IMMC). Cancer Epidemiol Biomarkers Prev. (2017) 26:876–85. 10.1158/1055-9965.EPI-16-0762-T 28223430 PMC5457306

[28] Marinac CR Birmann BM Lee IM Rosner BA Townsend MK Giovannucci E Body mass index throughout adulthood, physical activity, and risk of multiple myeloma: a prospective analysis in three large cohorts. Br J Cancer. (2018) 118:1013–9. 10.1038/s41416-018-0010-4 29527008 PMC5931105

[29] Hosgood HD Gunter MJ Murphy N Rohan TE Strickler HD. The relation of obesity-related hormonal and cytokine levels with multiple myeloma and non-hodgkin lymphoma. Front Oncol. (2018) 8:103. 10.3389/fonc.2018.00103 29713614 PMC5911620

[30] Nedal TMV Moen SH Roseth IA Tryggestad SS Aass KR Hov GG Diet-induced obesity reduces bone marrow T and B cells and promotes tumor progression in a transplantable Vk*MYC model of multiple myeloma. Sci Rep. (2024) 14:3643. 10.1038/s41598-024-54193-8 38351079 PMC10864380

[31] Choi T Kang Y. Chimeric antigen receptor (CAR) T-cell therapy for multiple myeloma. Pharmacol Ther. (2022) 232:108007. 10.1016/j.pharmthera.2021.108007 34582835 PMC8930424

[32] Shah UA Mailankody S. Emerging immunotherapies in multiple myeloma. Br Med J. (2020) 370:m3176. 10.1136/bmj.m3176 32958461

